# The retinal neurovascular coupling is impaired in men with vasculogenic erectile dysfunction

**DOI:** 10.1038/s41598-023-35339-6

**Published:** 2023-05-22

**Authors:** Enrico Borrelli, Alberto Quarta, Edoardo Pozzi, Giuseppe Fallara, Flavia Pennisi, Marco Casaluci, Francesca Lamanna, Lea Querques, Riccardo Sacconi, Francesco Bandello, Francesco Montorsi, Andrea Salonia, Giuseppe Querques

**Affiliations:** 1 Vita-Salute San Raffaele University, Via Olgettina 60, Milan, Italy; 2 Department of Ophthalmology, IRCCS San Raffaele Scientific Institute, Milan, Italy; 3 Division of Experimental Oncology/Unit of Urology, IRCCS San Raffaele Scientific Institute, Milan, Italy

**Keywords:** Diagnostic markers, Urology, Urogenital diseases, Eye diseases, Retinal diseases

## Abstract

The aim of this study was to study the retinal vessels in patients affected by vasculogenic erectile dysfunction (ED), using dynamic vessel analyzer (DVA). Patients with vasculogenic ED and control subjects were prospectively enrolled to undergo a complete urological and ophthalmologic evaluation, including DVA and structural optical coherence tomography (OCT). The main outcome measures were: (1) arterial dilation; (2) arterial constriction; (3) reaction amplitude (the difference between arterial dilation and constriction); and, (4) venous dilation. Thirty-five patients with ED and 30 male controls were included in the analysis. Mean ± SD age was 52.0 ± 10.8 years in the ED group and 48.1 ± 16.3 years in the control group (p = 0.317). In the dynamic analysis, the arterial dilation was lower in the ED group (1.88 ± 1.50%), as compared with the control group (3.70 ± 1.56%, p < 0.0001). Neither arterial constriction nor venous dilation differed between groups. The reaction amplitude was decreased in ED patients (2.40 ± 2.02%, p = 0.023), compared to controls (4.25 ± 2.20%). In the Pearson correlation analysis, the ED severity, was directly correlated with both reaction amplitude (R = .701, p = 0.004) and arterial dilation (R = .529, p = 0.042). In conclusion, subjects with vasculogenic ED are featured by a significant dysfunction of the retinal neurovascular coupling, which is inversely correlated with ED severity.

## Introduction

Erectile dysfunction (ED) represents a common disease in which subjects experience the incapacity to obtain and maintain an erection for satisfactory sexual performance^1^. This disorder may be featured by severe impact on the quality of life of patients and their partners^1,2^.

ED etiology has been historically subdivided into three main categories: (1) organic, (2) mixed, and (3) psychogenic^2^. Of note, although this classification has been widely employed to categorize patients, ED is a complex disorder with multifactorial factors involved. Therefore, terms “primary organic” or “primary psychogenic” have been implemented to redefine ED patients according to a binary etiological classification^1,2^. In primary organic ED, several factors have been involved in the evolution of this disease, including vascular, neurologic (i.e., caused by a deficit in nerve signaling to the corpora cavernosa), iatrogenic (e.g., secondary to radical pelvic surgery), and endocrinological factors. Among these, vasculogenic ED is the most common etiology as it accounts for most cases of primary organic ED^2^. In vasculogenic ED, an impairment of the proper functioning of the penile vasculature occurs, the latter requiring a complex interplay of multiple components.

Nitric oxide (NO) plays a major role in erectile function as this molecule is released from terminals of the cavernous nerves in the corpus cavernosum and activates guanylate cyclase in the cavernous smooth muscle cells, the latter process resulting in smooth muscle relaxation and arteriolar vasodilation^3^. Moreover, NO is also released from the vascular endothelium in response to parasympathetic activation^3^. Therefore, a neurovascular dysfunction in relation to an impairment in this NO-related process may lead to an impairment in blood flow regulation in the corpus cavernosum and finally contribute to the pathogenesis of vasculogenic ED.

Even though cavernous small vessel disease has been demonstrated to be involved in the pathogenesis of vasculogenic ED, the microcirculation in the corpus cavernosum is still difficult to be explored in vivo. Since the cavernous and retinal vasculature have resemblances in terms of morphologic and functional attributes, the assessment of the retinal vasculature may thus be beneficial to provide new relevant insights into the pathogenesis of vasculogenic ED.

Notably, a number of previous studies have already demonstrated that retinal vascular modifications may affect eyes of subjects with vasculogenic ED^4,5^. Nevertheless, all these reports have been limited to a morphological analysis, rather than a functional evaluation. Therefore, these reports were not able to provide an analysis on the retinal vessels’ NO-mediated processes that might be altered in men with vasculogenic ED.

Therefore, this study aimed at providing a quantitative assessment of the functional and morphological characteristics of the macular microvasculature in subjects affected by vasculogenic ED, using dynamic vessel analyzer (DVA) analysis. More importantly, we investigated associations between retinal vascular metrics and other clinical characteristics, including factors reflecting ED disease features and severity.

## Methods

### Study participants

In this observational and case–control study, non-Finnish, white-European men with a clinically confirmed diagnosis of vasculogenic erectile dysfunction were prospectively enrolled from the urology department at the IRCCS San Raffaele Hospital in Milan, Italy. The approval for this study was obtained by the San Raffaele Ethics Committee (NCT02845765 on clinicaltrials.gov) and a written informed consent was granted from all subjects prior to enrollment. This study adhered to the tenets of the Declaration of Helsinki.

**Figure 1:**
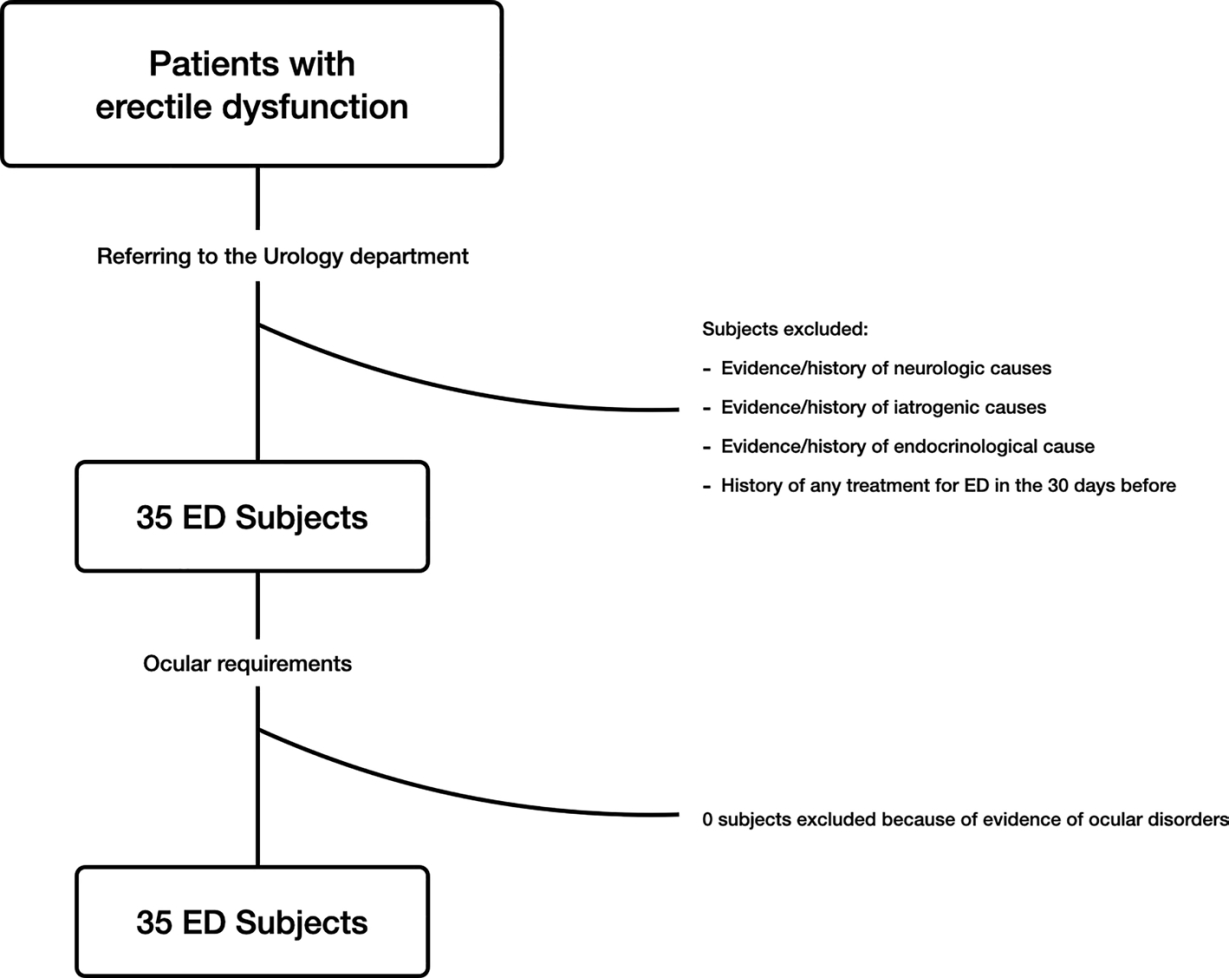
Flowchart diagram describing the selection process of eligible patients for this analysis. Among the initial cohort of subjects with erectile dysfunction (ED) who referred to the Urology department, only 35 patients were eligible for ophthalmological evaluation. None of these had ocular disorders which did not allow the enrollment.

All ED subjects underwent a standardized erectile function assessment before the enrollment, which included: (1) completion of the International Index of Erectile Function-Erectile Function domain (IIEF-EF) score, which is used to objectively quantify ED severity (i.e. lower values are associated with a more severe form of ED)^6^; (2) medical history collection in order to calculate the Charlson Comorbidity Index (CCI) score, which is the most extensively studied comorbidity index employed to measure comorbid disease status^7,8^; (3) body mass index (BMI) measurement, which was calculated as weight in kilograms by height in square meters, (4) blood tests, including the measurement of serum hormone levels in order to rule out endocrinological causes of ED; and, (5) dynamic penile color Doppler ultrasonography, that was performed in order to assess penile hemodynamics^1^. The latter examination allowed to obtain the cavernosal peak systolic velocity (PSV) and resistance index (RI) metrics (mean ± ED values between both corpora cavernosa). Exclusion criteria were the evidence and/or history of neurologic, iatrogenic, and endocrinological factors that may have resulted in primary organic non-vasculogenic ED. Furthermore, included patients had no history of any treatment for ED in the 30 days before enrollment (Fig. 1).

An age-matched control group of male volunteers with no evidence or history of ED was also included.

The following exclusion criteria were considered for either patient and control group: (1) history of uncontrolled chronic medical conditions, including systemic hypertension; (2) evidence of disease affecting the optic nerve or retina; (3) refractive error superior to 6 diopters (D); and (4) inability to obtain a pharmacologic pupillary dilation inferior to 6 mm.

The enrollment of patients and controls was completed between September 2018 and June 2021. Included subjects also underwent a complete ophthalmologic assessment, including measurement of best-corrected visual acuity (BCVA) and structural optical coherence tomography (OCT) and DVA imaging.

The SPECTRALIS HRA + OCT device (Heidelberg Engineering, Heidelberg, Germany) was employed to obtain structural OCT of the macula with a protocol of acquisition consisting in 19 horizontal B-scans. Successively, the built-in software was used to automatically segment and provide a thickness map of the different layers in the retina, as previously described^9,10^.

### Dynamic vessel analyzer

The DVA imaging was performed using a commercially available device (DVA; Imedos Systems UG, Jena, Germany), as previously described in detail^10–14^. This device is formed of a fundus camera, video camera, real-time monitor, and a personal computer with analysis software.

This device measures the retinal vessel modifications following diffuse luminance flicker provided to the retina (dynamic analysis). During the test, the examiner visualizes patients’ fundus with a red-free light while patients focus into the camera. Before the light stimulus, the examiner selects arteriolar and venular segments in order to record and measure their changes throughout the examination. The light flicker stimulation lasts 20 s and vessels changes are recorded and measured for 80 s. Notably, the system automatically suspends the test when subjects blink or move their eyes. Each eye underwent two cycles of stimulation and measurements.

Successively, the DVA system averaged the two obtained examinations for each eye in order to provide results on retinal arteriolar and venular changes in diameter in response to flickering light. These results were calculated as percentage (%) after comparison to baseline diameter values. Normal eyes are typically featured by a biphasic curve with a primary vasodilation and secondary vasoconstriction. The dynamic analysis provided the following metrics: (1) arterial dilation; (2) arterial constriction; (3) reaction amplitude (the difference between arterial dilation and constriction); and, (4) venous dilation.

Furthermore, the DVA device also provides a static analysis which measures the average diameter of all those arterioles and venules within a region of interest around the optic disc. The obtained metrics are: (1) central retinal artery equivalent (CRAE); (2) central retinal vein equivalent (CRVE); and, (3) artery–vein ratio (AVR—obtained by dividing CRVE by CRAE).

As the repeatability for DVA measurements have been reported previously to be good^15–18^, the reproducibility was not reassessed in the present study.

### Statistical analysis

The Statistical Package for Social Sciences (version 20.0, SPSS Inc., Chicago, IL, USA) was used for statistical analyses.

A Shapiro–Wilk’s test was performed to confirm a normal distribution for all the variables. Continuous variables were compared by conducting a Student T-test for independent variables or a one-way analysis of covariance (ANCOVA) with Bonferroni post-hoc test, by introducing potential confounding factors as covariates. Qualitative variables were compared using a Fisher’s exact test. Pearson’s chi-squared correlation was performed to evaluate the linear correlation between clinical factors reflecting ED dysfunction and retinal functional/anatomic metrics.

The sample size of the study was tested to be proper for a mean difference between groups of almost 10%, a power of 75% and type I error rate (α) of 5%.

A p value of 0.05 was considered for statistical significance.

## Results

### Characteristics of patients included in the analysis

We included 35 patients with ED and 30 age-matched controls. One eye for each subject was randomly selected and included in the analysis. Mean age was 52.0 ± 10.8 years [range 32–73 years] in the ED group and 48.1 ± 16.3 years [range 29–78 years] in the control group (p = 0.317). Mean ± SD duration of ED was 5.3 ± 3.7 months. The BCVA was 1.0 ± 0.0 LogMAR (Snellen VA of 20/20) in the ED group and 1.0 ± 0.0 LogMAR (Snellen VA of 20/21) in the healthy eyes (p = 1.0). Table 1 describes clinical characteristics of patients and controls.

**Table 1:** Characteristics of erectile dysfunction patients and controls.

	ED	Controls	*p* value
Number of eyes enrolled (patients)	35 (35)	30 (30)	–
Age (years)	52.0 ± 10.8	48.1 ± 16.3	0.317^a^
Smoke, n (%)	4 (11.43%)	2 (6.6%)	0.413^b^
Diabetes, n (%)	5 (14.28%)	0	0.039^b^
Hypertension, n (%)	12 (34.28)	0	< 0.0001^b^
Hypercholesterolemia, n (%)	5 (14.28%)	0	0.039^b^
BMI (kg/m^2^)	25.8 ± 2.7	24.3 ± 2.0	0.013^a^
Abdominal circumference (cm)	96.7 ± 10.3	92.9 ± 4.6	0.054^a^
Total blood testosterone (ng/mL)	4.9 ± 2.4	–	–
IIEF-EF	14.6 ± 7.7	–	–
CCI	0.34 ± 0.71	–	–
Age-adjusted CCI	2.0 ± 1.3	–	–
PSV (cm/s)	29.7 ± 9.7	–	–
RI	0.92 ± 0.07	–	–

^a^T-test.

^b^Fischer’s exact test.

Quantitative values are expressed in mean ± SD (standard deviation).

*ED* erectile dysfunction, *n* number of patients, *BMI* body mass index, *IIEF-EF* 6-item International Index of Erectile Function, *CCI* Charlson Comorbidity index, *PSV* peak systolic velocity, *RI* resistance index.

### Anatomic metrics

Optical coherence tomography results are presented in Supplemental Table 1. In the sectorial analysis, only the central OPL thickness was reduced in ED patients compared to controls (26.3 ± 4.6 µm versus 28.1 ± 2.7 µm, p = 0.020).

### Functional metrics—dynamic vessel analyzer

**Figure 2:**
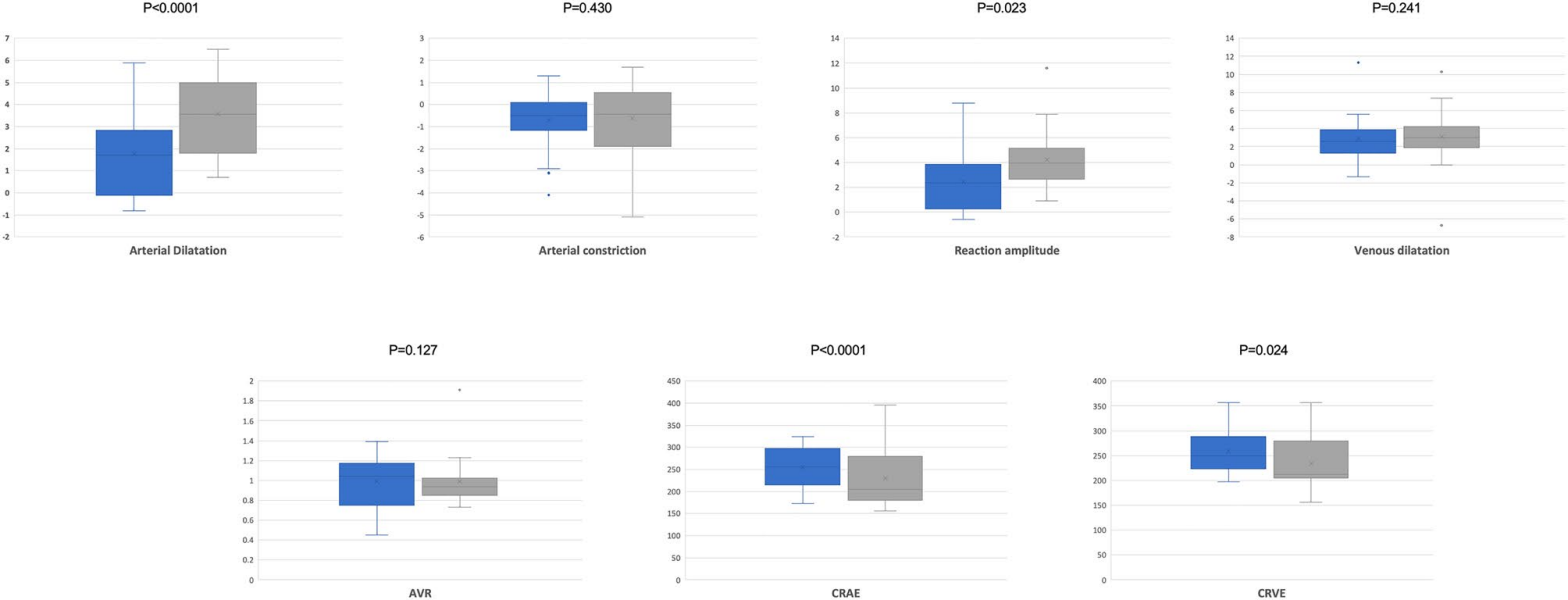
Box and whisker plots showing analyzed DVA measurements in patients and controls. Each box shows median (central horizontal line), mean (cross within the box) and interquartile range (horizontal extremes of the box) values for each variable. The ends of the whiskers represent the minimum and maximum values. Dots not included in whiskers represent outliers. Each graph (blue for ED patients and gray for healthy controls, respectively) shows values of a different metric in each of the two groups. P-values for each comparison are reported. Details on pairwise comparisons are presented in Table 2.

In the static analysis, both the CRAE (252.1 ± 47.4 mu in ED and 221.3 ± 56.6 mu in controls, p < 0.0001) and CRVE (259.3 ± 40.1 mu in ED and 229.7 ± 43.2 mu in controls, p = 0.024) were increased in ED patients (Fig. 2, Table 2).

**Table 2:** Dynamic vessel analyzer parameters in patients and controls.

	ED (N = 35)	Controls (N = 30)	p value
Dynamic analysis			
Arterial dilation (%)	1.88 ± 1.50	3.70 ± 1.56	< 0.0001
Arterial constriction (%)	− 0.56 ± 1.22	− 0.52 ± 1.54	0.430
Reaction amplitude (%)	2.40 ± 2.02	4.25 ± 2.20	0.023
Venous dilation (%)	2.79 ± 2.38	3.4 ± 2.96	0.241
Static analysis			
AVR	0.98 ± 0.27	0.97 ± 0.21	0.127
CRAE	252.1 ± 47.4	221.3 ± 56.6	< 0.0001
CRVE	259.3 ± 40.1	229.7 ± 43.2	0.024

Values are compared using a one-way analysis of covariance (ANCOVA) with age, diabetes, hypercholesterolemia and systemic hypertension as covariates. Data are presented as mean ± SD (standard deviation).

*N* number of patients, *ED* erectile dysfunction, *AVR* artery–vein ratio, *CRAE* central retinal artery equivalent, *CRVE* central retinal vein equivalent.

In the dynamic analysis, the arterial dilation was decreased in the ED group (1.88 ± 1.50%), in the comparison with the control group (3.70 ± 1.56%, p < 0.0001). Neither arterial constriction nor venous dilation differed between groups. The reaction amplitude was decreased in ED patients (2.40 ± 2.02%, p = 0.023), compared to controls (4.25 ± 2.20%) (Fig. 2, Table 2).

Supplemental Table 2 summarizes results on DVA after excluding patients with diabetes and/or systemic hypertension.

### Correlation analysis

In the Pearson correlation analysis, the CCI adjusted for age was correlated with the venous dilation (R = 0.350, p = 0.039) and AVR (R = -0.371, p = 0.036). The IIEF-EF score was directly correlated with both arterial dilation (R = 0.529, p = 0.042) and reaction amplitude (R = 0.701, p = 0.004). Other correlations are reported in Table 3.

**Table 3:** Pearson correlations in erectile dysfunction (ED) patients.

	Pearson coefficient (r)	p value
Arterial dilation (%)		
PSV (cm/s)	− 0.013	0.957
RI	0.230	0.330
CCI	0.053	0.776
CCI adjusted for age	0.130	0.458
IIEF-EF	0.529	0.042
Arterial constriction (%)		
PSV (cm/s)	0.423	0.063
RI	0.136	0.566
CCI	0.020	0.908
CCI adjusted for age	− 0.075	0.667
IIEF-EF	− 0.205	0.464
Reaction amplitude (%)		
PSV (cm/s)	− 0.253	0.282
RI	0.127	0.593
CCI	0.035	0.841
CCI adjusted for age	0.169	0.332
IIEF-EF	0.701	0.004
Venous dilation (%)		
PSV (cm/s)	0.292	0.211
RI	− 0.112	0.638
CCI	0.316	0.064
CCI adjusted for age	0.350	0.039
IIEF-EF	− 0.122	0.665
AVR		
PSV (cm/s)	− 0.423	0.090
RI	− 0.225	0.384
CCI	0.023	0.900
CCI adjusted for age	− 0.371	0.036
IIEF-EF	0.044	0.876
CRAE		
PSV (cm/s)	− 0.271	0.293
RI	0.075	0.776
CCI	0.015	0.936
CCI adjusted for age	− 0.218	0.230
IIEF-EF	0.057	0.841
CRVE		
PSV (cm/s)	0.349	0.169
RI	0.310	0.226
CCI	− 0.060	0.744
CCI adjusted for age	0.245	0.176
IIEF-EF	− 0.001	0.997

*AVR* artery–vein ratio, *CRAE* central retinal artery equivalent, *CRVE* central retinal vein equivalent, *PSV* systolic peak velocity, *RI* resistance index, *CCI* Charlson Comorbidity index.

## Discussion

In this report we provided a quantitative assessment of the retinal neurovascular coupling in eyes of subjects with primary organic vasculogenic erectile dysfunction. Overall, we showed that this physiological response to flicker stimulation is significantly affected in men with ED. Importantly, our results suggest that these modifications are significantly associated with ED severity, as scored with the IIEF-EF domain.

Even though ED is mainly considered as a disorder affecting the sexual activity, a number of evidences suggests that this disorder may be an indicator of systemic endothelial vascular disease. Previous evidences have indeed suggested that the presence of ED is associated with an increased risk of cardiovascular events^19,20^. Therefore, ED has been considered as an early manifestation of coronary artery and peripheral vascular disease^19,21,22^.

Furthermore, assuming that ED may imply a systemic endothelial dysfunction, previous studies have tried to elucidate whether these patients may be characterized by alterations in the retinal vessels. Using DVA, Chew et al.^5^ performed a static analysis of the retinal vessels in 106 patients with ED and type 2 diabetes with or without diabetic retinopathy. In agreement with our results, in the latter study^5^, the authors demonstrated that these patients are characterized by higher values of CRVE in comparison with healthy controls. Two hypotheses were suggested to explain this finding. Since a wider retinal venular diameter is thought to reflect inflammation^23^, it was supposed that a retinal venular dilatation may reflect a thickening of the intima-media secondary to atherosclerosis^5^. Alternatively, a wider retinal venular diameter may be secondary to endothelial dysfunction, as previously suggested^5,23^. Nevertheless, our findings are partially in disagreement with those reported by Chew et al.^5^, who showed a reduction in CRAE values in ED patients. However, in the study by Chew et al., enrolled patients were older than those analyzed in our study (i.e., mean age was 65.6 vs. 52.0 years) and a number of subjects had signs of diabetic retinopathy. Assuming that age and diabetic retinopathy are factors that were demonstrated to significantly affect CRAE values^23^, these study cohort differences between the two studies might explain such results’ inconsistencies. Importantly, our results of a wider retinal arteriolar diameter (i.e., increase in CRAE values) in ED patients may reflect a thickening of the intima-media^5^ or an endothelial dysfunction^5,23^.

We add to the literature by providing data on the retinal neurovascular coupling in subjects with primary organic vasculogenic erectile dysfunction. As asserted above, the DVA testing in healthy subjects provides a biphasic response reflecting a primary vasodilation and secondary vasoconstriction. The vasodilatation seems to be provoked by photoreceptors, the latter causing an increase in NO levels and subsequent rise in retinal blood flow^10,24^. In our ED cohort, the vessel response to the flicker stimulus was reduced and this reduction mainly characterized the arteries’ vasodilatation.

As asserted above, NO is released from the vascular endothelium and from terminals of the cavernous nerves and this results in smooth muscle relaxation and arteriolar vasodilation^3^. Therefore, an impairment in this NO-related process is thought to contribute to the pathogenesis of vasculogenic ED^25^. Similarly, our results may suggest that ED patients are also characterized by an impairment in the retinal vessels’ endothelium, with resulting diminished NO production, that is crucial for obtaining vasodilatation after the flicker stimulation. Alternatively, the smaller response might be secondary to an increased rigidity of the retinal arteries, in view of the fact that systemic vessels were displayed to be featured by a thickened intima-media in ED patients^23^. Finally, a decreased metabolic demand associated with neuronal degeneration might cause the lower vascular response. However, our structural OCT data did not show significant differences in terms of retinal structure between ED patients and healthy controls.

One of the most notable results from our study was that metrics reflecting the retinal neurovascular coupling (i.e., arterial dilation and reaction amplitude) were significantly associated with ED severity. In detail, we displayed that retinal arterial dilation and reaction amplitude are directly correlated with increases in IIEF-EF scores and improvements in erectile function. These results may further indicate a common pathophysiological process for ED and impaired retinal neurovascular coupling in these patients.

The main limitation of this report is that subjects were assessed at a single time point. A prospective study testing retinal vessels in ED patients may shed further light on whether an impaired retinal neurovascular coupling may also affect retinal function in these patients. Moreover, although patients in our study cohort were homogenously categorized as being affected by primary organic ED, we are not able to exclude that a psychogenic component may also have been present in a number of patients. Importantly, the latter aspect may have impacted on the IIEF-EF score. Furthermore, our study cohort was composed of Caucasian men, therefore our findings are limited to this kind of patients. Also, we did not provide data on measurements’ repeatability, although this was reported previously^15–18^. Finally, the sample size of our cohort is relatively small which reduces the power of our analysis. In particular, we were not powered to evaluate for small differences between the two groups.

In conclusion, this report provided a fully integrated assessment of either static and dynamic modifications in retinal vessels of subjects with primary organic vasculogenic erectile dysfunction. We showed that retinal vascular alterations distinguish this disorder and that the arterial function is inversely correlated with ED severity. Our results further confirm that ED may be associated with a systemic endothelial dysfunction. Future reports with prolongated longitudinal follow up of the studied cohort may furnish additional substantive information and retinal vascular metrics may prove to be a useful biomarker for monitoring the value of therapeutic approaches for ED or to predict the disease progression. More importantly, the assessment of retinal function in healthy men may provide information on whether these metrics may predict the development of ED over time.

## Supplementary Information

Supplementary Table 1.

Supplementary Table 2.

## Data Availability

The data used to support the findings of this study are available from the corresponding author upon request.
