# Supplementary material for: The retinal neurovascular coupling is impaired in men with vasculogenic erectile dysfunction
Source: Sci Rep. 2023 May 22;13:8237. doi: 10.1038/s41598-023-35339-6 (PMC10203266; doi:10.1038/s41598-023-35339-6)
Supplement: Supplementary file 1 — Supplementary Table 1. [file 41598_2023_35339_MOESM1_ESM.docx]

| Supplemental Table 1. Optical coherence tomography parameters in patients and controls. | | | |
| --- | --- | --- | --- |
| Region | **ED**  **(N=35)** | **Controls**  **(N=30)** | **P value** |
| Central total retinal thickness (µm) | 282.2±22.9 | 292.8±20.1 | 0.161 |
| Parafoveal total retinal thickness (µm) | 341.7±12.3 | 343.3±24.3 | 0.981 |
| Perifoveal total retinal thickness (µm) | 300.9±11.7 | 303.7±18.5 | 0.459 |
| Central RFLN thickness (µm) | 13.06±3.02 | 13.5±1.6 | 0.945 |
| Parafoveal RFLN thickness (µm) | 22.1±2.8 | 22.4±2.0 | 0.714 |
| Perifoveal RFLN thickness (µm) | 34.85±3.8 | 35.5±5.5 | 0.995 |
| Central GCL thickness (µm) | 17.0±6.4 | 18.2±4.5 | 0.660 |
| Parafoveal GCL thickness (µm) | 50.2±5.9 | 52.6±4.5 | 0.389 |
| Perifoveal GCL thickness (µm) | 36.6±3.1 | 37.0±4.6 | 0.154 |
| Central IPL thickness (µm) | 22.0±4.6 | 23.4±4.1 | 0.424 |
| Parafoveal IPL thickness (µm) | 42±3.1 | 43.5±2.5 | 0.276 |
| Perifoveal IPL thickness (µm) | 30.4±2.5 | 30.4±3.3 | 0.176 |
| Central INL thickness (µm) | 22.0±6.3 | 22.8±6.3 | 0.274 |
| Parafoveal INL thickness (µm) | 41.0±2.7 | 43.0±2.8 | 0.016 |
| Perifoveal INL thickness (µm) | 34.1±3.6 | 34.7±2.8 | 0.842 |
| Central OPL thickness (µm) | 26.3±4.6 | 28.1±2.7 | 0.020 |
| Parafoveal OPL thickness (µm) | 34.1±3.6 | 33.8±5.8 | 0.978 |
| Perifoveal OPL thickness (µm) | 28.6±2.3 | 27.5±3.1 | 0.083 |
| Central ONL thickness (µm) | 93.8±8.82 | 98.3±9.5 | 0.128 |
| Parafoveal ONL thickness (µm) | 70.2±7.2 | 70.6±7.2 | 0.800 |
| Perifoveal ONL thickness (µm) | 58.2±5.6 | 57.7±7.2 | 0.285 |
| Values are compared using a one-way analysis of covariance (ANCOVA) with age, diabetes, hypercholesterolemia and systemic hypertension as covariates. Data are presented as mean±SD (standard deviation).  N: number of patients; ED: Erectile dysfunction; RNFL: retinal nerve fiber layer; GCL: ganglion cell layer; IPL: inner plexiform layer; INL: inner nuclear layer; OPL: outer plexiform layer; ONL: outer nuclear layer. | | | |
