# Supplementary material for: The retinal neurovascular coupling is impaired in men with vasculogenic erectile dysfunction
Source: Sci Rep. 2023 May 22;13:8237. doi: 10.1038/s41598-023-35339-6 (PMC10203266; doi:10.1038/s41598-023-35339-6)
Supplement: Supplementary file 2 — Supplementary Table 2. [file 41598_2023_35339_MOESM2_ESM.docx]

| **Supplemental Table 2.** Dynamic vessel analyzer parameters in patients and controls. | | | |
| --- | --- | --- | --- |
|  | **ED**  **(N=27)** | **Controls**  **(N=30)** | **P value** |
|  |  |  |  |
| **Dynamic Analysis** |  |  |  |
| ***Arterial dilation*** *(%)* | 2.19±0.60 | 3.70±1.56 | <0.0001 |
| ***Arterial constriction*** *(%)* | -0.60±1.28 | -0.52±1.54 | 0.713 |
| ***Reaction amplitude*** *(%)* | 2.79±2.99 | 4.25±2.20 | 0.373 |
| ***Venous dilation*** *(%)* | 2.83±2.11 | 3.40±2.96 | 0.377 |
| **Static Analysis** |  |  |  |
| ***AVR*** | 0.99±0.25 | 0.97±0.21 | 0.112 |
| ***CRAE*** | 253.4±42.3 | 221.3±56.6 | <0.0001 |
| ***CRVE*** | 259.2±49.6 | 229.7±43.2 | 0.020 |
| Values are compared using a one-way analysis of covariance (ANCOVA) with age and hypercholesterolemia as covariates. Data are presented as mean±SD (standard deviation)  **N:** number of patients; **ED:** Erectile dysfunction; **AVR**: artery-vein ratio; **CRAE:** central retinal artery equivalent; **CRVE:** central retinal vein equivalent. | | | |
